# mtSTAT3 suppresses rheumatoid arthritis by regulating Th17 and synovial fibroblast inflammatory cell death with IL-17-mediated autophagy dysfunction

**DOI:** 10.1038/s12276-024-01376-y

**Published:** 2025-01-17

**Authors:** Seon-Yeong Lee, Jeonghyeon Moon, A Ram Lee, Young-Mee Moon, Jeong Won Choi, Chae Rim Lee, Su Been Jeon, Hee Su Sohn, Jeehee Youn, Dongyun Shin, Sung-Hwan Park, Mi-La Cho

**Affiliations:** 1 Lab of Translational ImmunoMedicine, Catholic Research Institute of Medical Science, College of Medicine, The Catholic University of Korea, Seoul, Republic of Korea; 2 Department of Pathology, College of Medicine, The Catholic University of Korea, Seoul, South Korea; 3 Departments of Neurology and Immunobiology, Yale School of Medicine, New Haven, CT USA; 4 Department of Biomedicine and Health Sciences, College of Medicine, The Catholic University of Korea, Seoul, Republic of Korea; 5 Division of Pulmonary and Critical Care Medicine, Department of Medicine, Brigham and Women’s Hospital, Boston, MA USA; 6 Harvard Medical School, Boston, MA USA; 7 Laboratory of Autoimmunology, Department of Anatomy and Cell Biology, Hanyang University College of Medicine, Seoul, Korea; 8 College of Pharmacy, Gachon University, Incheon, Republic of Korea; 9 Divison of Rheumatology, Department of Internal Medicine, Seoul St. Mary’s Hospital, College of Medicine, The Catholic University of Korea, Seoul, Republic of Korea

**Keywords:** Autoimmunity, Cell death and immune response, Rheumatoid arthritis

## Abstract

Th17 cells are activated by STAT3 factors in the nucleus, and these factors are correlated with the pathologic progression of rheumatoid arthritis (RA). Recent studies have demonstrated the presence of STAT3 in mitochondria, but its function is unclear. We investigated the novel role of mitochondrial STAT3 (mitoSTAT3) in Th17 cells and fibroblast-like synoviocytes (FLSs) and analyzed the correlation of mitoSTAT3 with RA. We used **a** collagen-induced arthritis (CIA) mouse model to determine the effect of mitochondrial STAT3. We observed changes in the RA mouse model via the use of a mitochondrial STAT3-inducing vector and inhibitor. We observed the accumulation of abnormal autophagosomes, increased inflammatory cell death signaling, and decreased mitoSTAT3 activity in FLSs from both patients with RA and patients with IL-17-treated FLSs. We first discovered that IL-17 increased the accumulation of abnormal autophagosomes and the expression of inflammatory cell death factors in synovial fibroblasts and decreased mitoSTAT3 activation. In a mouse model of CIA, arthritis and joint inflammation were decreased by injection vectors that induced mitoSTAT3 overexpression. The abnormal accumulation of autophagosomes and the expression of inflammatory cell death factors were also decreased in these mice. In mouse and human immune cells, ZnSO_4_, an inducer of mitochondrial STAT3, decreases the production of reactive oxygen species, the IL-17 concentration, and differentiation into Th17 cells. However, mitoSTAT3 blockade accelerated the development of arthritis, inflammatory cell death, and abnormal autophagosome/autophagolysosome formation. Therefore, this study suggests a novel inhibitory mechanism of RA using mitoSTAT3 via the regulation of autophagy, Th17 differentiation, and inflammatory cell death.

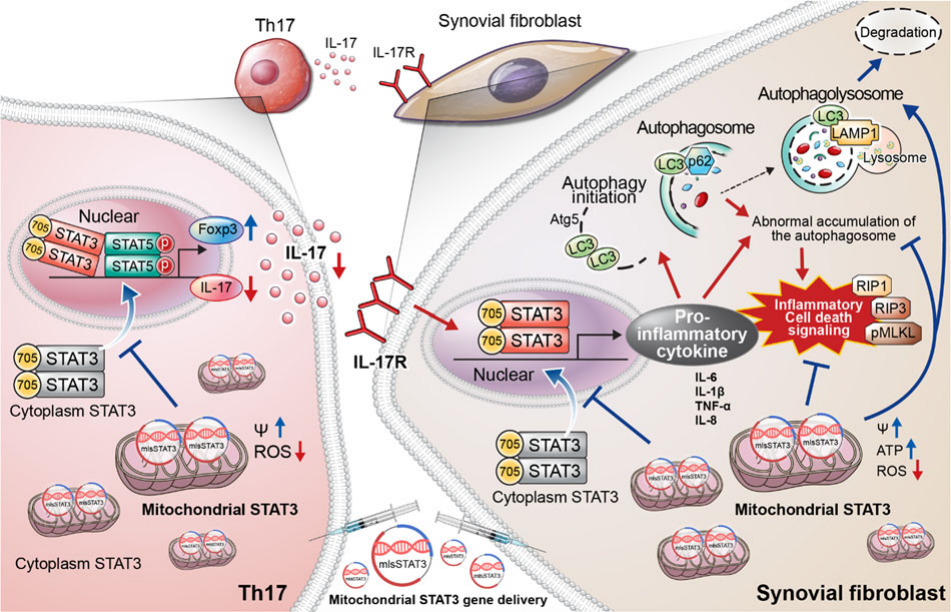

## Mitochondrial STAT3 modulates Th17 cells in rheumatoid arthritis

Rheumatoid arthritis is a long-term disease where the immune system attacks the joints, causing pain and swelling. Researchers have found that a protein called mitoSTAT3, located in the mitochondria, might help reduce inflammation in RA. Researchers used mice with arthritis and human cells to study mitoSTAT3’s role. They increased mitoSTAT3 levels in mice and observed less joint damage and inflammation and found that mitoSTAT3 helps control a process called autophagy. This is important because poor autophagy can worsen RA. The results showed that boosting mitoSTAT3 reduced inflammation and joint damage in mice. The researchers concluded that mitoSTAT3 could be a new target for RA treatment by improving cell cleanup processes and reducing harmful immune responses. In the future, therapies that increase mitoSTAT3 might help people with RA by reducing inflammation and joint damage.

This summary was initially drafted using artificial intelligence, then revised and fact-checked by the author.

## Introduction

Rheumatoid arthritis (RA) is a chronic autoimmune disease characterized by immune dysregulation that causes inflammation, swelling, and pain in multiple joints^1^. Excessive activation of T helper 17 (Th17) cells, which predominantly produce interleukin (IL)-17, is considered the primary factor responsible for the pathogenesis of RA^2,3^. Additionally, IL-17 and granulocyte‒macrophage colony‒stimulating factor secreted by Th17 cells induce macrophage activation, and IL-17, IL-22, and IL-23 secreted by Th17 cells stimulate synovial fibroblasts to express IL-1β, IL-6, and matrix metalloproteinase-1, recruiting macrophages into joints^4–6^. This combined reaction leads to synovial inflammation^7^. Therefore, the regulation of Th17 cells is critical in the treatment of RA.

The development of Th17 cells requires the activity of signal transducer and activator of transcription 3 (STAT3)^8^. STAT3 is activated by proinflammatory cytokines, such as interferon (IFN), IL-5, and IL-6^9,10^. Activation begins with the phosphorylation of tyrosine 705 (Y705) by Janus kinases (JAKs), which are associated with receptors stimulated by cytokines. Following this phosphorylation, the activated complex translocates to the nucleus, where it triggers the expression of various genes, such as IL-21, IL-22, and IL-17^11,12^. Activated phosphorylated STAT3^Y705^ (*p*-STAT3^Y705^) induces the differentiation of Th17 cells, which are involved in various autoimmune diseases, including RA^13^. Recent studies have also shown that *p*-STAT3^Y705^ induces homodimerization of STAT3, nuclear translocation, DNA binding, and downstream gene expression (including the expression of IL-6, IL-8, IL-17, IL-22, and the RAR-related orphan receptor γt)^14,15^. In contrast to *p*-STAT3^Y705^, however, the phosphorylation of STAT3 at serine 727 (Ser^727^; *p*-STAT3^S727^) leads to the translocation of STAT3 into the mitochondria^16,17^. Although the role of *p*-STAT3^S727^ is unclear, various activation signals, such as extracellular signal-regulated kinases 1 and 2, p38, and mitogen-activated protein kinase^18,19^^,^ have been shown to be involved in the Ser^727^ phosphorylation of STAT3. Mitochondrial *p*-STAT3^S727^ and nuclear/cytoplasmic *p*-STAT3^Y705^ have conflicting functions^14,20^. The JAK pathway induces *p*-STAT3^Y705^ to enter the nucleus, but genes associated with retinoid–IFN-induced mortality-19 (GRIM-19) recruit STAT3 to the mitochondria and induce *p*-STAT3^S727^. Mitochondrial STAT3 (mitoSTAT3) promotes mitochondrial respiration by interacting with mitochondrial proteins and contributes to the inflammatory response through stimulation of immune cell migration and cytokine secretion^21,22^. Hence, the modulation of mitoSTAT3 may serve as a novel target in the treatment of inflammatory diseases because of its effects on the immune response. We have published studies showing that GRIM-19 regulates inflammation and alleviates symptoms in an autoimmune disease model^23–25^; however, we have not elucidated the mechanism underlying how GRIM-19 enhances the function of mitochondria and regulates gene expression. In the present study, we focused on determining the function of mitoSTAT3 transformed by GRIM-19.

Interestingly, both the IL-17-treated synovial fibroblasts and the synovial fibroblasts of patients with RA presented abnormally high levels of autophagosome accumulation. Autophagy is an evolutionarily conserved process that isolates nonessential intracellular components for lysosomal degradation in response to various stresses, including deprivation of nutrients or growth factors, hypoxia, reactive oxygen species (ROS) accumulation, DNA damage, irregular protein aggregation, and damage to organelles^26,27^. Autophagy affects not only the development, homeostasis, survival, and differentiation of immune cells but also the pathogenesis of autoimmune diseases^28,29^.

Following normal and complete autophagy, LC3-II remains linked to the autophagosome membrane^30^. Lysosomes are recruited to autophagosomes and their cargo for fusion and subsequent degradation^31^. Lysosomes contain lysosome-associated membrane proteins 1 and 2 (LAMP-1 and -2, respectively), which are required for lysosome trafficking and fusion with autophagosomes^32^. After autophagosome–lysosome fusion, degradation may occur depending on the acidic pH inside the lysosome^33^.

When autophagosome–lysosome fusion normally occurs, the cargo and p62 protein in the autophagosome are degraded by the acidic environment^34^. Chemical agents such as bafilomycin and chloroquine inhibit autophagy completion by blocking autophagosome‒lysosome fusion^35^, leading to the accumulation of autophagosomes and residual p62 protein within cells. In previous studies, abnormal autophagosome accumulation was observed in patients with sepsis or an induced inflammatory response^36,37^. Therefore, we hypothesized that IL-17 stimulation leads to the accumulation of intracellular debris through abnormal autophagosome accumulation in synovial fibroblasts, promoting the inflammatory response.

Taken together, these novel findings suggest for the first time that mitoSTAT3 effectively inhibits the pathogenesis of RA in contrast to nuclear STAT3.

## Methods

### Animals

Six-week-old male DBA/1J (SLC, Inc., Shizuoka, Japan), C57BL/6 (Jackson Laboratory), BALB/c (ORIENT Bio), and pEG-mitochondrial localization sequence-STAT3-FLAG (MLS-STAT3) transgenic (TG) mice were kept in cages in a specific pathogen-free environment. All experimental procedures were reviewed and approved by the Animal Research Ethics Committee of the Catholic University of Korea (CUMC-2020-0048-02).

### Type II collagen-induced arthritis (CIA)

To induce arthritis, DBA1/J mice were administered type II collagen and complete Freund’s adjuvant (Chondrex) into the tail. Two weeks after immunization, the mice were boosted with type II collagen and incomplete Freund’s adjuvant (Chondrex). One week after CIA induction, the pEG-MLS-stopGFP Mock, pEG-MLS-mSTAT3-FLAG (MLS-STAT3), pRc/CMV-FLAG Mock, or pRc/CMV-STAT3^Y705F^-FLAG (705 mutant) vector was injected weekly via hydrodynamic injection for 7–9 weeks via electroporation. A description of the construction of the plasmid vector and collagen antibody-induced arthritis are presented in the Supplementary Methods. To test the efficacy of mitoSTAT3 inhibition^32^, the inhibitor was fed to the mice daily after the first immunization. The severity of arthritis was recorded via the arthritis index (range: 0–4)^38^.

### Western blot

Whole protein and extracted mitochondrial protein samples were separated via 10% sodium dodecyl sulfate–polyacrylamide gel electrophoresis and transferred to a nitrocellulose membrane (Amersham Pharmacia Biotech). Primary antibodies against RIP1, RIP3, *p*-MLKL, *p*-STAT3^S727^, *p*-STAT3^Y705^, STAT3, Mfn2, DRP1, *p*-DRP1, LC3, p62, GAPDH, tubulin, Cox4, or β-actin (Cell Signaling Technology) were diluted at a concentration of 1:500–1:1000. After washing, secondary antibodies were added, and the membrane was incubated for 15 min at room temperature. The signal was detected via an enhanced chemiluminescence detection kit and Hyperfilm-ECL reagents (Amersham Pharmacia Biotech). The cell culture conditions are described in the Supplementary Methods.

### Cytokine enzyme-linked immunosorbent assay (ELISA)

The concentrations of IL-17 and IL-10 in the culture supernatant were measured via human or mouse ELISA kits (R&D Systems), and the absorbance was analyzed at 450 nm via a microplate reader (Molecular Devices).

Serum was collected from mice injected with mock or MLS-STAT3 vectors 9 weeks after arthritis induction. IgG and IgG2a were measured via specific ELISA kits (Bethyl Laboratories) according to the manufacturer’s protocol. The absorbance was analyzed at 450 nm via a microplate reader (Molecular Devices).

### Flow cytometry

To analyze Th17 and T regulatory cells (Tregs), ex vivo splenocytes, cultured splenocytes, or peripheral blood mononuclear cells were stained with specific anti-CD4-peridinin-chlorophyll protein and/or anti-mouse CD25-allophycocyanin (APC) antibodies for 30 min at 4 °C. These cells were permeabilized and fixed with Cytoperm/Cytofix (BD Biosciences) for Th1, Th2, and Th17 cells and eBioscience™ Foxp3/Transcription Factor Staining Buffer (Invitrogen) for Tregs in accordance with the manufacturer’s protocols. The cells were further stained with fluorescein-5-isothiocyanate (FITC)-conjugated anti-IL-17, anti-IFN-γ-PE, and IL-4-APC (Invitrogen) for Th1, Th2, and Th17 cells, respectively. Phycoerythrin (PE)-conjugated anti-Foxp3 was used for Treg staining for 30 min at 4 °C. Then, the washed cells were subjected to flow cytometry (Calibur; BD Biosciences).

### Immunohistopathological analysis

The fixed and decalcified tissues were sliced to a thickness of 7 µm, dewaxed with xylene, dehydrated with different concentrations of alcohol, and stained with hematoxylin and eosin (H&E), safranin O, and toluidine blue to detect proteoglycans. The H&E-stained sections were evaluated for inflammation and bone erosion^38^. Immunohistochemistry was performed using a Vectastain ABC kit (Vector Laboratories). Joint sections were incubated with specific antibodies (against IL-17, IL-6, TNF-α, IL-1β, RIP1, RIP3, and *p*-MLKL; Abcam) overnight at 4 °C. The tissues were incubated with biotinylated secondary antibodies with a streptavidin‒peroxidase complex for 1 h. Immunohistochemistry was performed on tissue sections from all the mice (*n* = 5) in both groups. Three slides were prepared for each mouse, and the slides were taken at least 500 μm apart. Immunostained sections were examined via a photomicroscope (Olympus). The number of positive cells per high-power field (magnification: 400×) was counted via Adobe Photoshop software (Adobe), and the average of three randomly selected fields per tissue section was recorded.

### Immunofluorescence imaging

RA fibroblast-like synoviocytes (FLSs) or spleen tissues were obtained 7 or 9 weeks after CIA induction for confocal microscopy. To identify autophagosomes and autophagolysosomes, the tissues were stained with anti-LC3-FITC, anti-p62-PE, anti-LAMP1-PE, and DAPI. To analyze immune cells, mitochondria and mitoSTAT3, the tissues were stained with anti-CD4-FITC, anti-IL-17-PE, anti-Foxp3-PE, anti-CD25-APC, anti-*p*-STAT3-727-PE and anti-Cox4-APC (eBioscience) antibodies. The stained sections were analyzed via a confocal microscopy system (LSM 510-Meta; Carl Zeiss) and ZEN blue edition (Carl Zeiss).

### Synthesis of mitoSTAT3 inhibitors

The chemical that inhibits mitoSTAT3 was provided by Professor DY Shin (Gachon University, South Korea). The inhibitors were synthesized based on the results of a previous study^32^.

### Statistical analysis

Statistical analyses were performed via IBM SPSS Statistics software, version 20.0 (IBM). Two-sided *p* values of <0.05 were considered statistically significant. One- and two-way analysis of variance was used for comparisons of multiple groups. The Bonferroni post hoc correction was applied to data with statistically significant differences in the analysis of variance. Numerical data were compared between groups via the nonparametric Mann–Whitney *U* test (two-tailed).

## Results

### IL-17 and mitoSTAT3 regulate autophagy initiation and inflammatory cell death

**Fig. 1:**
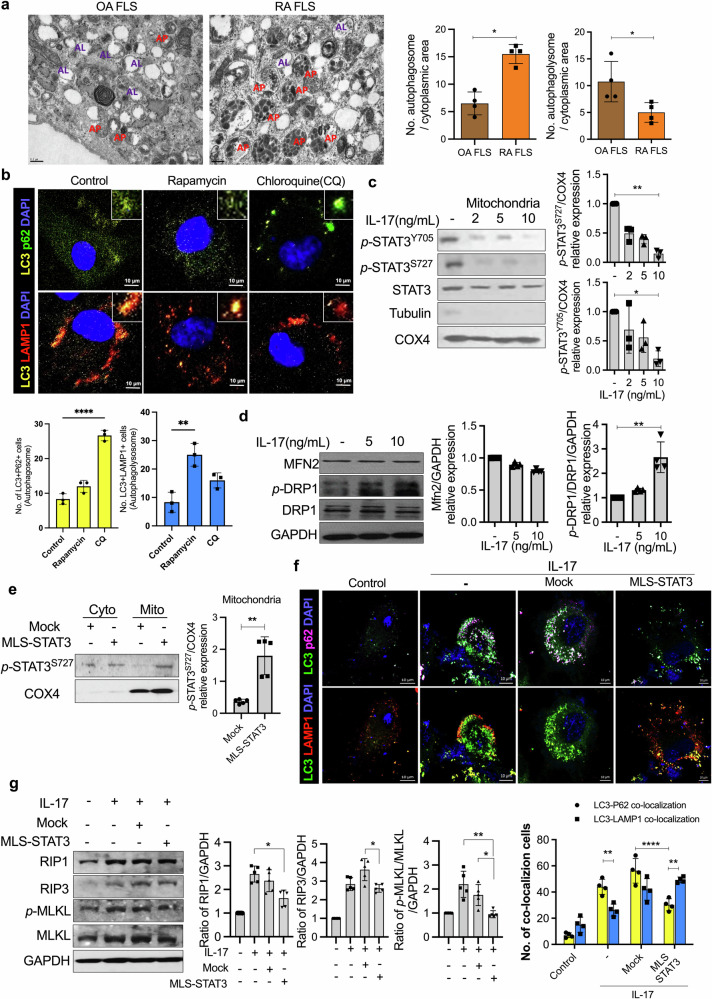
IL-17 suppressed mitoSTAT3 in RA FLSs and induced mitochondrial dysfunction, inflammatory cell death, and autophagosome accumulation. **a** Autophagosomes and autophagolysosomes were analyzed in OA and RA FLSs via transmission electron microscopy (scale bars = 0.5 μm, AP autophagosome, AL autophagolysosome). **b** RA FLSs were cultured with 10 μM rapamycin and 20 μM chloroquine for 24 h. Autophagosomes and autophagolysosomes were analyzed with anti-LC3-APC antibodies, anti-p62-FITC antibodies, anti-LAMP1-PE antibodies, and DAPI via confocal microscopy. **c** Mitochondria were isolated from IL-17-stimulated RA FLSs. The mitochondrial lysates were analyzed via western blotting with anti-*p-*STAT3^Y705^, anti-*p-*STAT3^S727^, anti-STAT3, anti-tubulin, and anti-COX4 antibodies. **d** RA FLSs were cultured with IL-17 for 12 h and then lysed to obtain proteins. The expression levels of MFN2, DRP1, and *p-*DRP1, which are dynamic mitochondrial molecules, were measured. **e** RA FLSs were transfected with mock or MLS-STAT3 vectors. *P-*STAT3^S727^ expression in cytoplasmic lysates and mitochondrial lysates was analyzed via western blotting. **f** RA FLSs were transfected with mock or MLS-STAT3 DNA vectors. The cells were cultured with IL-17 for 24 h posttransfection. Autophagosomes and autophagolysosomes were analyzed via confocal microscopy using anti-LC3-APC, anti-p62-FITC, and anti-LAMP1-PE antibodies and DAPI. **g** RA FLSs were cultured with IL-17 (10 ng/mL) for 24 h posttransfection. Inflammatory cell death molecules in the cell lysates were analyzed by western blotting. All the experiments were performed in triplicate. Bars represent the mean ± standard deviation (*p < 0.05, **p < 0.01, ***p < 0.005, ****p < 0.001).

In a previous study, we observed abnormal mitochondrial function and autophagosome formation in RA FLSs. Therefore, we analyzed autophagy and mitochondria formation via transmission electron microscopy of FLSs from patients with RA and patients with osteoarthritis (OA). Transmission electron microscopy images revealed the accumulation of autophagosomes in RA FLSs as well as less autophagosome–lysosome fusion in RA FLSs than in OA FLSs (Fig. 1a). This accumulation of autophagosomes (LC3-p62 colocalization) was like that observed after treatment with 20 μM chloroquine. On the other hand, the number of autophagolysosome fusions (LC3-LAMP1 colocalizations) increased after treatment with 10 μM rapamycin (an autophagy inducer that improves autophagosome–lysosome fusion) (Fig. 1b).

Not only are mitochondria and autophagy closely related, but mitochondrial dysfunction induced by chronic inflammation is also closely related to RA^39^. Therefore, we hypothesized that the accumulation of autophagosomes in patients with RA is associated with chronic inflammation, such as that in IL-17-abundant conditions, through mitochondrial modulation. To identify the relationships among mitochondria, IL-17, and autophagosome accumulation, the FLSs of patients with RA were treated with IL-17. We observed a decrease in *p*-STAT3 in IL-17-treated mitochondria (Fig. 1c). COX4 was used as a mitochondrial marker. In addition, IL-17 increased the expression of *p*-DRP1, which is a marker of mitochondrial fission (Fig. 1d).

We hypothesized that these phenomena were caused by reduced mitoSTAT3. Therefore, a vector capable of overexpressing mitoSTAT3 was constructed and transfected into RA FLS; the results verified that the level of STAT3 in mitochondria significantly increased (Fig. 1e). Importantly, abnormal autophagosome accumulation was observed in IL-17-treated RA FLSs. The colocalization of autophagosomes (LC3-p62) was detected more frequently than that of autophagolysosomes (LC3-LAMP1) following treatment with IL-17. However, MLS-STAT3 injection increased autophagolysosome formation even with IL-17 treatment (Fig. 1f).

Mitochondrial functions, including autophagy and cell death, are dynamic. Next, we studied the role of mitoSTAT3 in inflammatory cell death signaling. To determine whether IL-17 signals elicit the death of RA FLSs, we treated RA FLSs with 10 ng/mL IL-17 and quantified the levels of inflammatory cell death markers (Supplementary Fig. 1). Among the inflammatory cell death markers, RIP1, RIP3, and *p*-MLKL were significantly increased after IL-17 treatment. Moreover, RIP1, RIP3, and *p*-MLKL were significantly decreased by MLS-STAT3 transfection (Fig. 1g).

Overall, IL-17 increased the accumulation of autophagosomes, mitochondrial dysfunction (data not shown)^40^, and inflammatory cell death and decreased mitoSTAT3. However, the overexpression of mitoSTAT3 resolved the irregular accumulation of autophagosomes and reduced inflammatory cell death signaling. Therefore, these data suggest that the mechanism of mitoSTAT3 is different from that of nuclear/cytoplasmic STAT3 and that mitoSTAT3 can be used in RA treatment to regulate autophagy and mitochondria.

### Systemic mitoSTAT3 overexpression reduces the severity of arthritis in CIA mice

**Fig. 2:**
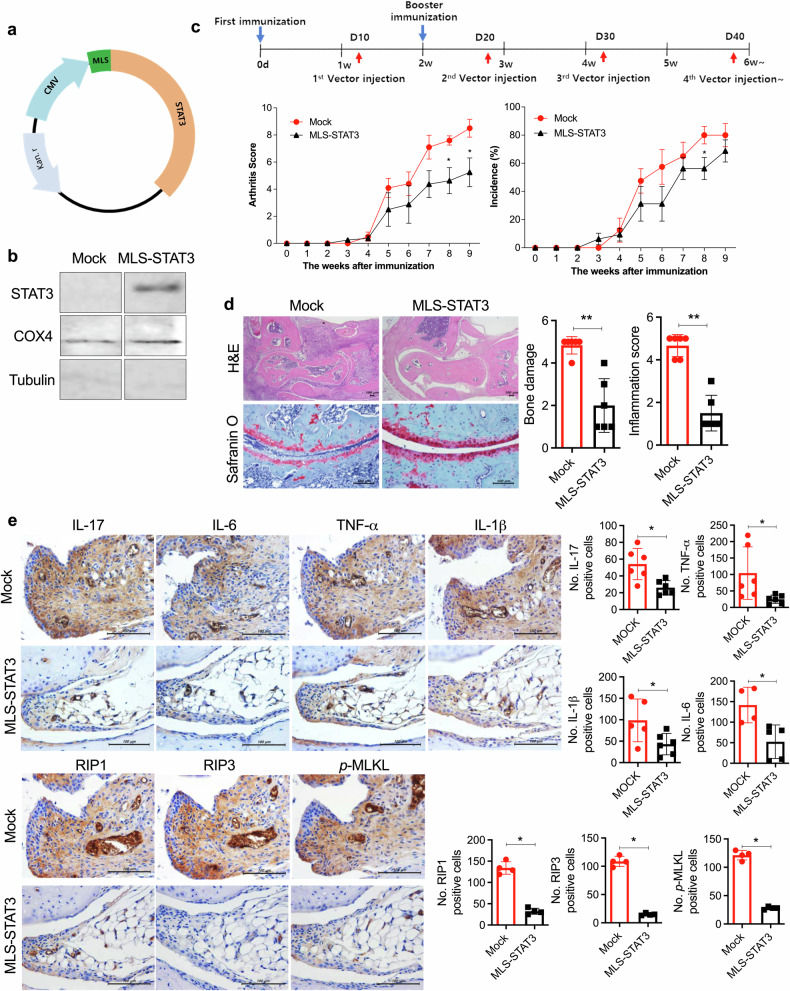
mitoSTAT3 suppressed the pathological changes associated with arthritis in CIA-affected mice. **a** Schematic map of the mouse MLS-STAT3-FLAG vector. **b** Western blot of mitochondria from mock- and MLS-STAT3-transfected NIH3T3 cells. Lanes were derived from the same gel and rearranged. **c** Schematic map of the schedule for arthritis induction. A total of 100 μg of mock or MLS-STAT3 DNA vector was injected every 10 days for 9 weeks after the first immunization. The severity scores and incidence of arthritis were analyzed every week. **d**, **e** Ankle joints were obtained 9 weeks after the first immunization, and the tissues were stained with H&E or antibodies against IL-17, IL-6, TNF-α, IL-1β, RIP1, RIP3, or *p*-MLKL. Representative images (scale bar = 100 μm) are displayed as the number of positive cells (dark brown) in five mice from each group. All the experiments were performed in triplicate. Arthritis scores are displayed as the mean ± standard error of the mean, and the bars represent the mean ± standard deviation (*p < 0.05, **p < 0.01).

To determine whether increased mitoSTAT3 expression has a therapeutic effect on autoimmune diseases, we delivered the mitoSTAT3 overexpression vector into mice with CIA. We designed a mitoSTAT3 overexpression vector using a cytomegalovirus (CMV) promoter and MLS near the STAT3 sequence (Fig. 2a). This vector allows overexpressed STAT3 to migrate into mitochondria. The expression of mitochondrial STAT3 was confirmed in MLS-STAT3-transfected cells (Fig. 2b) and the splenic tissue of mice (Supplementary Fig. 2).

We transferred MLS-STAT3 or the mock vector into mice with CIA every 10 days after the first immunization. The arthritis score and incidence were monitored every week, and serologic and histopathological examinations were carried out after the mice were sacrificed following the completion of six injections. The arthritis score and incidence were decreased in MLS-STAT3 vector-injected mice with CIA (Fig. 2c). Histopathological examination revealed that the histopathological scores, which represent the extent of inflammation and the degree of cartilage damage, were significantly lower in MLS-STAT3 vector-injected mice than in control mice (Fig. 2d). Proinflammatory cytokines and inflammatory cell death markers were evaluated via immunohistochemical examination of joint synovial tissues. The expression of proinflammatory cytokines (IL-17, IL-6, TNF-α, and IL-1β) and inflammatory cell death markers (RIP1, RIP3, and *p*-MLKL) was increased in the joint synovial tissues of the mice with CIA, whereas the expression of these markers was reduced in the joint synovial tissues of the MLS-STAT3 vector-injected mice (Fig. 2e). These results demonstrated that increased mitoSTAT3 expression mediated arthritis, inflammation, and cell death in this in vivo mouse model.

### Elevated mitoSTAT3 expression regulated Th17 differentiation, inflammatory cell death, and autophagosome accumulation

**Fig. 3:**
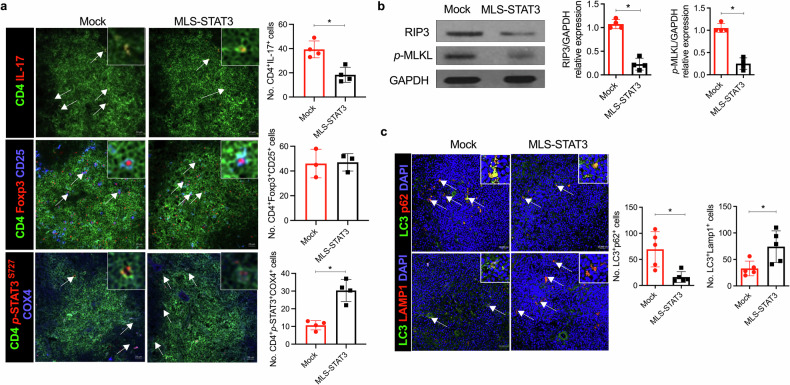
mitoSTAT3 regulated the Th17 cell population, inflammatory cell death, and autophagosome accumulation. **a**–**c** Mock- or MLS-STAT3-injected mice were sacrificed at 9 weeks after the first immunization. **a** Splenic tissues were stained with specific antibodies for the analysis of Th17 cells (anti-IL-17-PE and anti-CD4-FITC), Tregs (anti-Foxp3-PE, anti-CD25-APC, and anti-CD4-FITC), and mitochondrial STAT3 (anti-*p*-STAT3^S727^-PE, anti-CD4-FITC, and anti-Cox4-APC). Representative numbers of positive cells from five different tissues are shown (left). **b** The expression levels of RIP3, *p-*MLKL, and GAPDH were measured in splenocyte lysates from the mice. **c** Splenic tissues were stained with anti-LC3-FITC, anti-p62-PE, and anti-LAMP1-PE antibodies and DAPI for autophagosome and autophagolysosome analysis. Bars represent the mean ± standard deviation (*p < 0.05).

To investigate whether mitoSTAT3 overexpression regulates the population of immune cells in mice with CIA, we analyzed immune cells via immunofluorescence and western blotting. We found that Th17 cells (CD4^+^IL-17^+^) were decreased and mitoSTAT3-positive CD4^+^ cells (CD4^+^COX4^+^*p*-STAT3^+^) were increased by MLS-STAT3 vector injection. In contrast, the MLS-STAT3 overexpression vector did not affect the number of Foxp3^+^ Tregs (Fig. 3a). In addition, the levels of inflammatory cell death proteins were decreased in splenocytes after MLS-STAT3 vector injection (Fig. 3b). Interestingly, the fusion of autophagosomes and lysosomes, as evidenced by LC3-LAMP1 colocalization, was increased in MLS-STAT3 vector-injected mice with CIA, whereas the number of LC3^+^p62^+^ cells exhibiting autophagosome–lysosome uncoupling was decreased (Fig. 3c).

These results demonstrated that mitoSTAT3 regulates not only T-cell homeostasis and inflammatory cell death but also autophagosome–autophagolysosome formation. Thus, these findings suggest that the overexpression of mitoSTAT3 regulates the systemic immune system and autophagy.

### Overexpression of mitoSTAT3 ameliorated arthritis under severe inflammatory conditions via the STAT3^Y705^ mutant vector

**Fig. 4:**
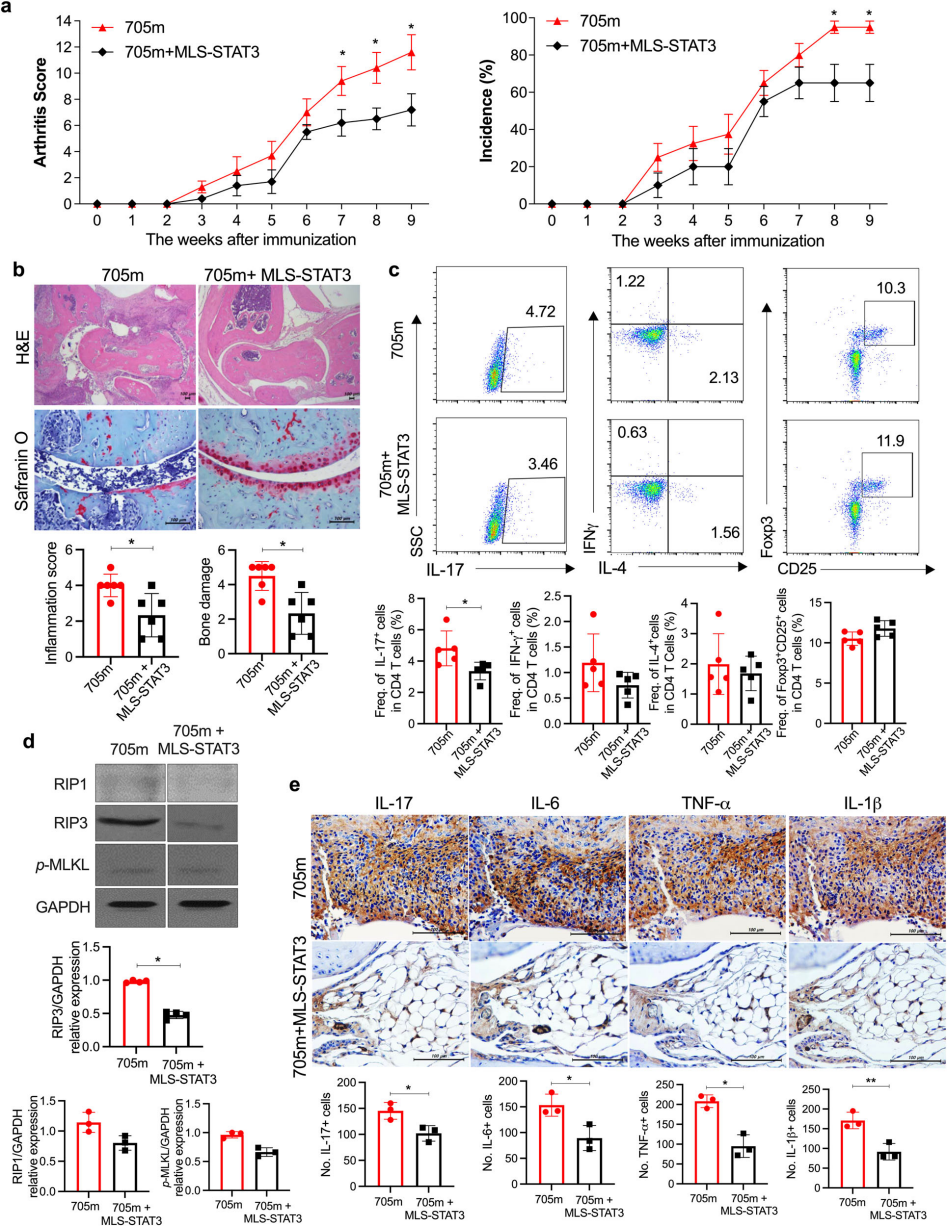
Activation of cytoplasmic STAT3 induced the development of arthritis. **a**–**e** 100 μg of the 705 mutant (STAT3^Y705F^) or the 705 mutant or the MLS-STAT3 DNA vector was injected every 10 days for 9 weeks after the first immunization. **a** Severity scores and incidence of arthritis were analyzed every week. **b** Ankle joints were obtained at 9 weeks after the first immunization, and the tissues were stained with H&E and safranin O for analysis of inflammation and bone damage. **c** Populations of IFN-γ^+^, IL-4^+^, IL-17^+^, and CD25^+^Foxp3^+^ cells among splenic CD4^+^ T cells were analyzed by flow cytometry. **d** The expression levels of RIP1, RIP3, *p-*MLKL, and GAPDH were measured in splenocyte lysates from each group. Lanes were derived from the same gel and rearranged. **e** Ankle joint tissues were stained with antibodies against IL-17, IL-6, TNF-α, and IL-1β. Representative images (scale bar = 100 μm) are displayed as the number of positive cells (dark brown) in three mice from each group. All the experiments were performed in triplicate. Arthritis scores are displayed as the mean ± standard error of the mean, and the bars represent the mean ± standard deviation (*p < 0.05, **p < 0.01).

High expression of nuclear/cytoplasmic STAT3 is observed in many patients with autoimmune diseases. For this reason, to increase the effect of p-STAT3^Y705^, we injected a nuclear/cytoplasmic p-STAT3^Y705^ mutant vector (STAT3^Y705F^; 705 m) with or without MLS-STAT3 into mice with CIA to mimic the chronic inflammatory situation in patients with RA^41^. CIA-affected mice with elevated STAT3 expression presented a more severe inflammatory response than did the general CIA-affected mice used in the previous experiment. In parallel, in MLS-STAT3 705 m vector-injected mice, mitoSTAT3 expression inhibited the inflammatory response more strongly under high-inflammatory conditions than under normal conditions. The severity and incidence of arthritis were significantly decreased in MLS-STAT3 vector-injected mice (Fig. 4a). H&E-stained and safranin O-stained images revealed joint tissue damage. Bone and cartilage damage was significantly less severe in MLS-STAT3 vector-injected mice than in control mice even when nuclear/cytoplasmic STAT3 was overexpressed (Fig. 4b). Murine splenocytes were analyzed by flow cytometry, which revealed that the Th17 cell population was significantly decreased by MLS-STAT3 vector injection. The numbers of Th1, Th2, and Treg cells did not notably differ between the 705 m group and 705 m with MLS-STAT3 vector-injected group (Fig. 4c). Moreover, the protein levels of RIP1, RIP3, and p-MLKL in murine splenocytes were reduced by mitoSTAT3 overexpression (Fig. 4d). The immunohistochemical images revealed that the overexpression of mitoSTAT3 decreased the expression of proinflammatory cytokines such as IL-17, IL-6, TNF-α, and IL-1β in the joint synovial tissues of CIA-affected mice (Fig. 4e). These data suggest that mitoSTAT3 regulates inflammation, disease progression, and inflammatory cell death in severe inflammatory conditions induced by cytoplasmic STAT3 overexpression.

### mitoSTAT3 inhibited the differentiation of Th17 cells in murine and human immune cells

**Fig. 5:**
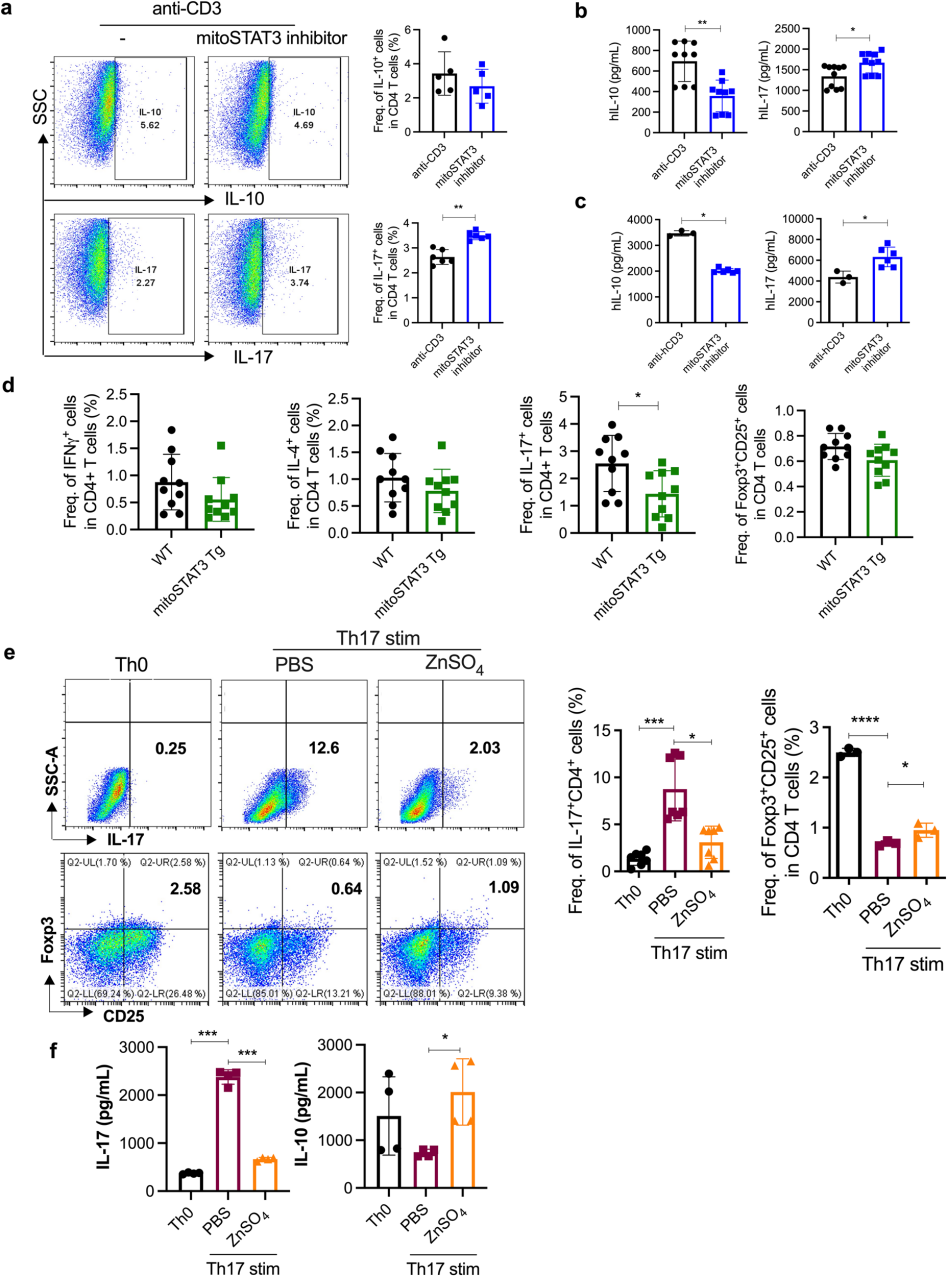
mitoSTAT3 altered Th17 differentiation by regulating mitochondrial function in mouse and human cells. **a**, **b** Healthy peripheral blood mononuclear cells were cultured under T-cell activation conditions in the presence or absence of a mitoSTAT3 inhibitor (0.1 μM). Three days later, the cells were stained with antibodies against CD4, IL-17, and IL-10 and analyzed via flow cytometry. The concentrations of IL-17 and IL-10 were measured via ELISA. **c** Human RA synovial fluid mononuclear cells were cultured with an anti-CD3 antibody in the presence or absence of a mitoSTAT3 inhibitor. The concentrations of IL-17 and IL-10 were measured via ELISA. **d** Th1, Th2, Th17 and Treg cells were analyzed by flow cytometry in splenocytes from mitoSTAT3 TG mice. **e**, **f** Splenic CD4^+^ T cells from C57BL/6 mice were cultured under Th17-inducing conditions in the presence or absence of ZnSO_4_ (10 μM). **e** At 3 days poststimulation, the cells were stained with antibodies against CD4, IL-17, CD25, and Foxp3 and analyzed via flow cytometry. **f** Concentrations of IL-17 and IL-10 in culture supernatants were measured via ELISA. All the experiments were performed in triplicate. The bars represent the mean ± standard deviation (*p < 0.05, **p < 0.01, ***p < 0.005, ****p < 0.001).

**Fig. 6:**
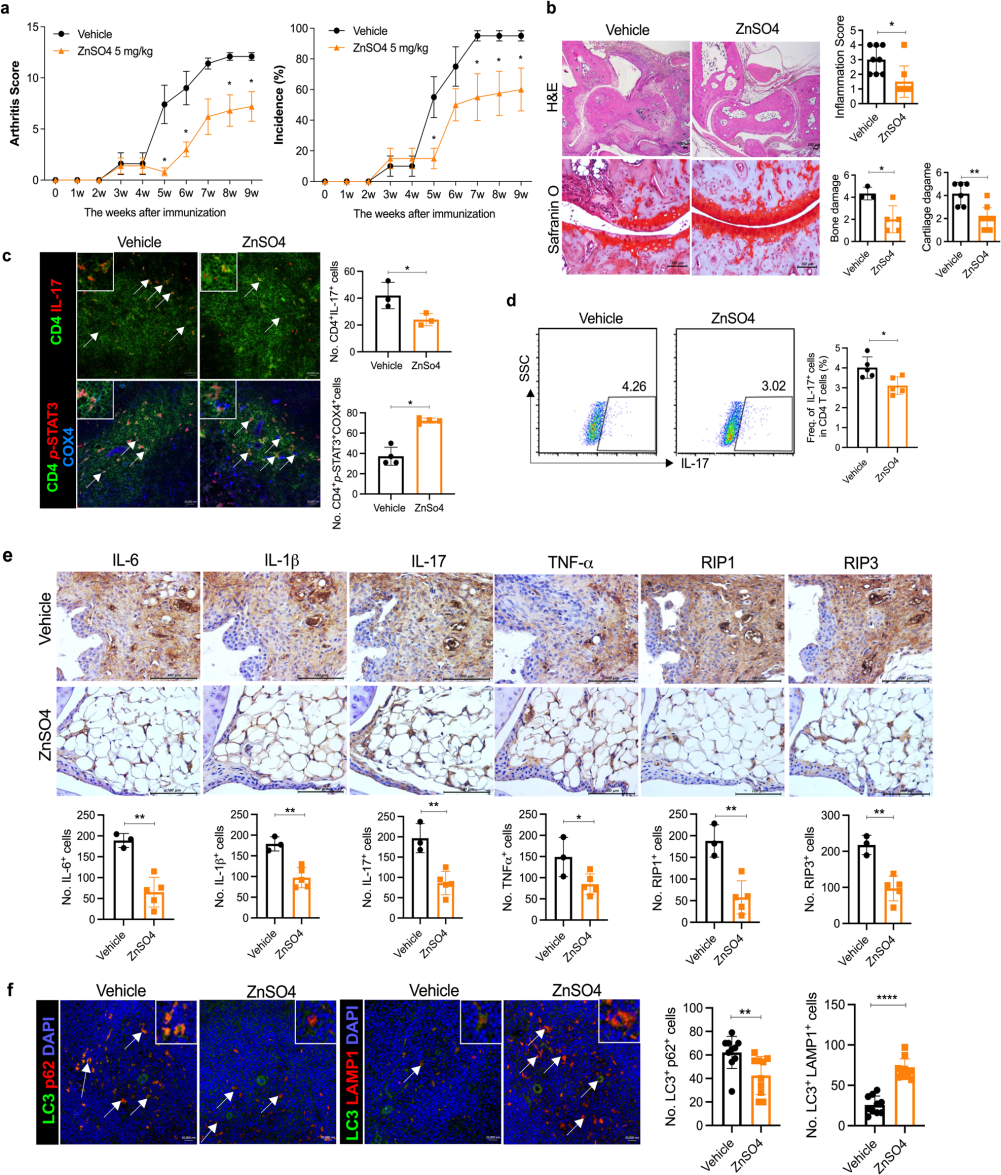
ZnSO_4_, a mitoSTAT3 inducer, alleviated the pathogenesis of arthritis through T-cell homeostasis. **a**–**f** Seven days after CIA induction, ZnSO_4_ (5 mg/kg) was orally administered for 9 weeks. **a** Severity scores and incidence of arthritis were analyzed every week. **b** Ankle joints were obtained at 9 weeks, and the tissues were stained with H&E and safranin O for analysis of inflammation and bone damage. **c** Splenic tissues were stained with specific antibodies for the analysis of Th17 cells (anti-IL-17-PE and anti-CD4-FITC) and mitochondrial STAT3 (anti-*p*-STAT3^S727^-PE, anti-CD4-FITC, and anti-Cox4-APC). **d** Splenocytes were stained with anti-CD4 and anti-IL-17 antibodies at 9 weeks after the first immunization and analyzed by flow cytometry. **e** The sectioned ankle joint tissues were immunohistochemically stained to identify cells positive for IL-6, IL-1β, IL-17, TNF-α, RIP1, and RIP3. Representative images (scale bar = 100 μm) are displayed as the number of positive cells (dark brown) in five mice per group. **f** Splenic tissues were stained with anti-LC3-FITC, anti-p62-PE, and anti-LAMP1-PE antibodies and DAPI for autophagosome and autophagolysosome analysis. Arthritis scores are displayed as the mean ± standard error of the mean, and the bars represent the mean ± standard deviation (*p < 0.05, **p < 0.01, ****p < 0.001).

Next, we synthesized a 600-molecular-weight mitoSTAT3 blocking chemical to inhibit mitoSTAT3. To investigate whether mitoSTAT3 directly regulates Th17 cells, we subjected human and murine immune cells to mitoSTAT3 blockade. We confirmed that the mitoSTAT3 inhibitor prevents STAT3 expression in the mitochondria of human OA FLS (Supplementary Fig. 3). When healthy peripheral blood mononuclear cells were treated with mitoSTAT3 blockade, the number of IL-17^+^CD4^+^ T cells significantly increased (Fig. 5a). In addition, the level of IL-10 secretion decreased, whereas that of IL-17 increased after mitoSTAT3 blockade (Fig. 5b). Next, synovial fluid mononuclear cells from patients with RA were cultured with or without mitoSTAT3 blockers, and the supernatant was analyzed via ELISA. The secretion of IL-17 was increased, whereas that of IL-10 was decreased by mitoSTAT3 treatment (Fig. 5c). We also studied mitoSTAT3-upregulated TG mice (mitoSTAT3 Tg). Th17 and mitochondrial ROS were significantly decreased in mitoSTAT3 Tg mice (Fig. 5d and Supplementary Fig. 4a–c). To investigate the effects of elevated mitoSTAT3 on immune cells, we treated murine splenocytes with ZnSO_4_. ZnSO_4_ most efficiently increased mitochondrial STAT3, and its safety was verified by oral administration; thus, we used ZnSO_4_ in subsequent experiments (Supplementary Fig. 5). In mice, Th17 differentiation was significantly decreased by ZnSO_4_ treatment under in vitro Th17 differentiation conditions (Fig. 5e). In contrast, the number of Foxp3^+^ Tregs was notably increased in the ZnSO_4_-treated group. ZnSO_4_ decreased the production of IL-17 and increased that of IL-10 during Th17 differentiation (Fig. 5f). In addition, compared with depolarized mitochondria, ZnSO_4_ reduced the ratio of healthy mitochondria to depolarized mitochondria, as shown by JC-1 staining (Supplementary Fig. 4d), and mitochondrial ROS, as shown by MitoSOX staining (Supplementary Fig. 4e). Next, we used ZnSO_4_, which is believed to be a mitoSTAT3 inducer in previous in vitro experiments and applied it to an RA mouse model. We orally administered 5 mg/kg ZnSO_4_ to CIA-affected mice for 9 weeks. The clinical arthritis score and incidence were significantly decreased by ZnSO_4_ treatment (Fig. 6a). H&E-stained and safranin O-stained images revealed the suppressive effect of ZnSO_4_ on inflammation and damage to bone and cartilage (Fig. 6b). Immunofluorescence revealed decreased IL-17^+^CD4^+^ cells and increased COX4^+^p-STAT3^+^CD4^+^ cells in mouse splenic tissues. The number of IL-17^+^CD4^+^ cells was significantly decreased, and the number of *p*-STAT3^+^COX4^+^CD4^+^ cells was increased by ZnSO_4_ treatment (Fig. 6c). Additionally, the number of Th17 cells within splenocytes was reduced by ZnSO_4_ treatment, as shown by flow cytometry (Fig. 6d). Immunohistochemistry revealed that the expression of proinflammatory cytokines (IL-6, IL-1β, IL-17, and TNF-α) and inflammatory cell death markers (RIP1 and RIP3) in mouse joint tissues was decreased by ZnSO_4_ treatment (Fig. 6e). In addition, the number of autophagosomes (LC3-p62) was decreased, and the number of autophagolysosomes (LC3-LAMP1) was increased in ZnSO_4_-treated mice with CIA (Fig. 6f). Therefore, an increase in mitoSTAT3 via ZnSO_4_ treatment has therapeutic effects on RA through a decrease in proinflammatory cytokines and inflammatory cell death markers combined with an increase in autophagosome–lysosome fusion.

### mitoSTAT3 blockade exacerbated arthritis in CIA-affected mice through increased IL-17 expression

**Fig. 7:**
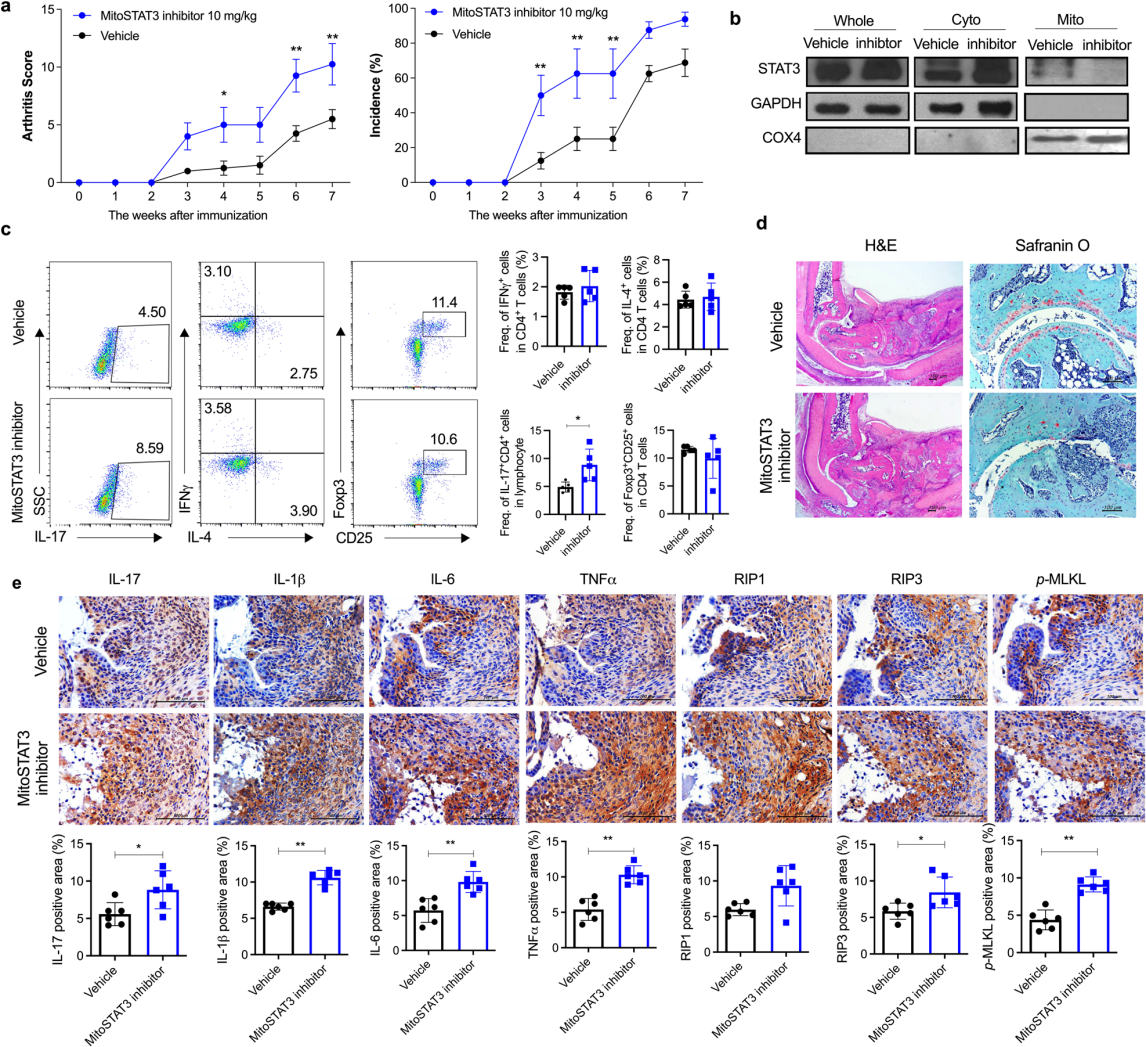
mitoSTAT3 blockade exacerbated arthritis pathology in CIA-affected mice through increased IL-17 expression. **a**–**e**, Seven days after CIA induction, a mitoSTAT3 inhibitor (10 mg/kg) was intraperitoneally injected into CIA-affected mice 3 times weekly. Joint and spleen tissues were obtained 7 weeks after the first immunization. **a** Severity scores and incidence of arthritis were analyzed every week. **b** Western blot data demonstrating the expression of mitoSTAT3 in splenocytes from mitoSTAT3 inhibitor-injected or vehicle-injected mice with CIA. Lanes were derived from the same gel and rearranged. **c** Splenocytes were stained with anti-CD4, anti-IL-17, anti-IFN-γ, anti-IL-4, anti-CD25, and anti-Foxp3 antibodies. **d**, **e** Sectioned ankle joint tissues were stained with H&E and safranin O and immunohistochemically stained with antibodies against IL-17, IL-1β, IL-6, TNF-α, RIP1, RIP3, and *p*-MLKL. All the experiments were performed in triplicate. Arthritis scores are displayed as the mean ± standard error of the mean, and the bars represent the mean ± standard deviation (*p < 0.05, **p < 0.01).

We demonstrated that mitoSTAT3 has therapeutic effects on arthritis through the regulation of immune cells and the autophagy pathway and that the inhibition of mitoSTAT3 increases Th17 differentiation, as shown by previous in vitro data (Fig. 5). To verify whether mitoSTAT3 inhibition leads to severe arthritis in the in vivo murine RA model, we intraperitoneally injected 10 mg/kg mitoSTAT3 inhibitor into CIA-affected mice 3 times weekly. The mitoSTAT3 inhibitor increased joint swelling and the incidence of arthritis in CIA-affected mice (Fig. 7a). Next, to determine whether mitoSTAT3 blockade specifically inhibits mitoSTAT3 in vivo, protein expression was analyzed via western blotting. There were no notable differences in the expression of STAT3 in splenocytes between the group treated with vehicle and the group treated with mitoSTAT3 blockade; however, STAT3 expression in mitochondria was decreased by the mitoSTAT3 inhibitor (Fig. 7b). Additionally, the number of Th17 cells in splenocytes was dramatically increased by treatment with the mitoSTAT3 inhibitor (Fig. 7c). To confirm the damage to the joint tissues, the tissues were stained with H&E and safranin O. H&E and safranin O staining revealed that joint damage and cartilage disruption were increased by mitoSTAT3 blockade injection (Fig. 7d). In addition, immunohistochemical analysis of synovial tissues revealed that the expression of IL-17, IL-1β, IL-6, and TNF-α was also increased by mitoSTAT3 inhibition. Moreover, RIP1, RIP3, and *p*-MLKL were increased in mitoSTAT3-inhibited mice (Fig. 7e). These results demonstrate that mitoSTAT3 inhibition plays a critical role in RA and that its downregulation leads to severe RA symptoms.

## Discussion

Proinflammatory cytokines such as IL-17, IL-6, TNF-α, and IL-1β are involved in the development of RA^42,43^. In particular, IL-17 plays a key role in the progression of RA^10^; thus, the regulation of IL-17 is important in RA treatment^44^. In this study, we evaluated a novel strategy to treat RA by using mitoSTAT3 to reduce the IL-17 level through the regulation of mitochondrial function and autophagy.

First, we discovered an interesting phenomenon in patients with RA in this study. Compared with those from patients with OA, synovial fibroblasts from patients with RA presented increased levels of autophagosome accumulation, whereas autophagolysosome levels were markedly lower than those in patients with OA. This finding is like the results of treatment with IL-17 or the autophagy inhibitor chloroquine. The accumulation of autophagosomes within the lymphocytes and granulocytes of patients with RA has been shown to be correlated with disease activity^45^. Chloroquine is known to be a drug that interferes with the combination of autophagosomes and lysosomes. In this study, we confirmed that, like chloroquine, IL-17 inhibits the binding of autophagosomes and lysosomes. This process inhibits the completion of autophagy and induces inflammation through the accumulation of autophagosomes.

In contrast to chloroquine, rapamycin (an mTORC1 blockade agent) induces autophagy by promoting lysosome–autophagosome fusion. Both chloroquine and rapamycin have been used to treat RA, and chloroquine has been used to block autoantigen processing in antigen-presenting cells but has shown no practical treatment effect^46^. In contrast, rapamycin promotes autophagy, inhibits osteoclasts, and inhibits proinflammatory cytokines, making it useful in the treatment of RA. In addition, mitophagy and mitochondria are linked to the inflammatory response. Additionally, we found that IL-17 increased the level of the phosphorylated form of Drp1, a mitochondrial fission marker^47^. When excessive mitochondrial fission occurs, the level of ROS increases, which is consistent with the previous finding that RA has high levels of ROS^48^. In addition, mitochondrial fission helps facilitate mitophagy, which involves the breakdown and recycling of damaged mitochondria^49^. The abnormal mitochondrial fission and mitophagy caused by IL-17 may be related to the abnormal autophagy process we observed. Hence, we hypothesized that abnormal autophagy may lead to excessive inflammation and inflammatory cell death and focused on autophagy and mitochondria for RA treatment in this study.

In RA, CD4^+^ T cells and FLSs exhibit reduced mitochondrial membrane potential^50^. Furthermore, the mitoSTAT3 pathway is known to regulate lymphocyte function^51^. Mitochondrial dysfunction regulates JAK1-STAT1/3 through the LKB1-AMPK pathway^52^, suggesting an interaction between mitochondrial function and JAK/STAT. Therefore, previous studies have suggested that mitochondria and autophagy dysregulation are involved in the progression of RA. In the present study, we demonstrated that increased mitoSTAT3 regulated both autophagy and Th17 differentiation. Furthermore, increased mitoSTAT3 expression reduced the severity of arthritis in CIA-affected mice.

Previous studies have shown that mitoSTAT3 has anti-inflammatory effects that differ from those of nuclear/cytoplasmic STAT3^53,54^. To determine whether mitoSTAT3 overexpression can cure RA, we used a mitoSTAT3-overexpressing vector with a CMV promoter and an MLS vector to treat RA-affected mice. MLS-STAT3 cannot induce all the STAT3 inserted into the vector to phosphorylate S727, but previous studies have shown that MLS-STAT3 clearly increases the phosphorylation of S727 (PMID: 24019511). Compared with those of control mice, the progression of RA, joint tissue damage, immune cell infiltration, and the secretion of inflammatory cytokines such as IL-17, IL-6, TNF-α, and IL-1β were inhibited in mitoSTAT3-overexpressing mice. Additionally, the expression of inflammatory cell death markers (RIP1, RIP3, and *p*-MLKL) was decreased. Therefore, mitoSTAT3 reduces the severity of arthritis and regulates the systemic immune response. Flow cytometry and immunofluorescence data revealed reduced numbers of Th17 cells among the splenocytes of mice injected with the mitoSTAT3-overexpressing vector.

Nuclear/cytoplasmic STAT3 signaling acts as a major intrinsic pathway for inflammation^55,56^. The activation of nuclear/cytoplasmic STAT3 plays a crucial role in the regulation of many genes related to inflammation through the induction of IL-17, IL-6, IL-22, MCP-1, IRF4, and RAR-related orphan receptor γt^57^. Therefore, we hypothesized that elevated nuclear/cytoplasmic STAT3 exacerbates RA progression and inflammation and tested whether increasing mitoSTAT3 can ameliorate disease progression. We injected the *p*-STAT3^S727^-overexpressing vector into CIA-affected mice to induce nuclear/cytoplasmic STAT3 expression. In parallel, a mitoSTAT3-overexpressing vector was also injected to increase the level of nuclear/cytoplasmic STAT3. As a result, the mice overexpressing nuclear/cytoplasmic STAT3 had more severe arthritis (mean arthritis score: 11.6 ± 4.2) than did the control mice (mean arthritis score: 8.5 ± 2.0) (Fig. 2). The overexpression of mitoSTAT3 prevented the progression of RA and suppressed joint inflammation and inflammatory cell death even under conditions of intensified inflammation through nuclear/cytoplasmic STAT3 overexpression.

The application of mitoSTAT3 overexpression gene therapy in patients in the clinical setting is challenging. Therefore, we used ZnSO_4_, which is known to induce mitoSTAT3, and observed that ZnSO_4_ reduced inflammation by improving mitochondrial function^58–60^. ZnSO_4_ inhibited the differentiation of Th17 cells under in vitro Th17-promoting conditions and inhibited the in vivo development of arthritis in mice. ZnSO_4_ reduced the severity of arthritis, the expression of inflammatory cytokines, and the number of systemic Th17 cells and regulated autophagosome–autophagolysosome formation.

Finally, to identify the role of mitoSTAT3 under conditions in which mitoSTAT3 is decreased, we synthesized a mitoSTAT3 inhibitor. Arthritis worsened, and joint damage and inflammatory cell death increased in the mitoSTAT3 inhibitor-treated mice.

S727-phosphorylated STAT3 appears to play a critical role in its presence in mitochondria. However, it cannot be asserted that S727 phosphorylation is the definitive cause, as its role is completely different from that of Y705 phosphorylation. It is presumed that the location of STAT3 plays a greater role in distinguishing its role as a nuclear transcription factor or mitochondrial function regulator than does its phosphorylation pattern. Many studies have investigated the role of STAT3; however, most have focused on the role of nuclear STAT3. Biological research on its phosphorylation pattern began relatively recently, so data concerning STAT3 phosphorylation are still lacking. Therefore, the role of the minor form, S727-phosphorylated STAT3, needs to be further studied.

Taken together, the results of this study indicate that mitoSTAT3 reduces inflammation in both animal models of RA and human fibroblasts. Our data suggest that the role of STAT3 depends on its phosphorylation pattern and location. Although further studies are needed to fully understand the detailed mechanism of action, IL-17 leads to the accumulation of autophagosomes in human fibroblasts, like FLSs in patients with RA. Although we did not identify the molecules controlled by mitoSTAT3 in this study, mitoSTAT3 was shown to reduce autophagosome accumulation and inflammation. We verified the effects of mitoSTAT3 in various conditions and animals via the use of a mitoSTAT3-overexpressing vector, nuclear/cytoplasmic STAT3 activation, a mitoSTAT3 enhancer, and a mitoSTAT3 inhibitor. Additionally, these data show that an increase in mitoSTAT3 may help patients with RA recover by improving mitochondrial function. Because mitochondrial dysfunction has been reported in many patients with RA, improving this dysfunction via mitoSTAT3 may in turn improve the symptoms of RA. Therefore, mitoSTAT3 plays a critical role in human FLSs through the regulation of autophagy and inflammatory cell death.

## Supplementary information

Supplementary Information

## References

[1] Guo, Q. et al. Rheumatoid arthritis: pathological mechanisms and modern pharmacologic therapies. *Bone Res.* **6**, 15, 10.1038/s41413-018-0016-9 (2018). 29736302 10.1038/s41413-018-0016-9 PMC5920070

[2] Golebski, K. et al. IL-1beta, IL-23, and TGF-beta drive plasticity of human ILC2s towards IL-17-producing ILCs in nasal inflammation. *Nat. Commun.* **10**, 2162, 10.1038/s41467-019-09883-7 (2019). 31089134 10.1038/s41467-019-09883-7 PMC6517442

[3] Yang, C. et al. Altered CD4+ T cell and cytokine levels in peripheral blood and skin samples from systemic sclerosis patients and IL-35 in CD4+ T cell growth. *Rheumatology* **61**, 794–805, 10.1093/rheumatology/keab359 (2022). 33878182 10.1093/rheumatology/keab359

[4] Dong, C. Genetic controls of Th17 cell differentiation and plasticity. *Exp. Mol. Med.* **43**, 1–6, 10.3858/emm.2011.43.1.007 (2011). 21270506 10.3858/emm.2011.43.1.007 PMC3041933

[5] Hata, H. et al. Distinct contribution of IL-6, TNF-alpha, IL-1, and IL-10 to T cell-mediated spontaneous autoimmune arthritis in mice. *J. Clin. Invest.* **114**, 582–588, 10.1172/JCI21795 (2004). 15314695 10.1172/JCI21795 PMC503774

[6] Inada, M. & Krane, S. M. Targeting rheumatoid inflammation and joint destruction in the mouse. *J. Clin. Invest.* **110**, 611–612, 10.1172/JCI16549 (2002). 12208860 10.1172/JCI16549 PMC151118

[7] Rosenblum, M. D., Remedios, K. A. & Abbas, A. K. Mechanisms of human autoimmunity. *J. Clin. Invest.* **125**, 2228–2233, 10.1172/JCI78088 (2015). 25893595 10.1172/JCI78088 PMC4518692

[8] Park, M. J. et al. Establishment of a humanized animal model of systemic sclerosis in which T helper-17 cells from patients with systemic sclerosis infiltrate and cause fibrosis in the lungs and skin. *Exp. Mol. Med.* **54**, 1577–1585, 10.1038/s12276-022-00860-7 (2022). 36175484 10.1038/s12276-022-00860-7 PMC9534900

[9] Chaudhry, A. et al. CD4+ regulatory T cells control TH17 responses in a Stat3-dependent manner. *Science* **326**, 986–991, 10.1126/science.1172702 (2009). 19797626 10.1126/science.1172702 PMC4408196

[10] Milner, J. D. et al. Impaired T(H)17 cell differentiation in subjects with autosomal dominant hyper-IgE syndrome. *Nature* **452**, 773–776, 10.1038/nature06764 (2008). 18337720 10.1038/nature06764 PMC2864108

[11] Brennan, F. M. & McInnes, I. B. Evidence that cytokines play a role in rheumatoid arthritis. *J. Clin. Invest.* **118**, 3537–3545, 10.1172/JCI36389 (2008). 18982160 10.1172/JCI36389 PMC2575731

[12] Ren, J. et al. Serum- and glucocorticoid-inducible kinase 1 promotes alternative macrophage polarization and restrains inflammation through FoxO1 and STAT3 signaling. *J. Immunol.* **207**, 268–280, 10.4049/jimmunol.2001455 (2021). 34162726 10.4049/jimmunol.2001455 PMC8695641

[13] Miao, J. et al. CD147 expressed on memory CD4(+) T cells limits Th17 responses in patients with rheumatoid arthritis. *Front. Immunol.* **11**, 545980, 10.3389/fimmu.2020.545980 (2020). 33193313 10.3389/fimmu.2020.545980 PMC7655988

[14] Cheng, X., Peuckert, C. & Wolfl, S. Essential role of mitochondrial Stat3 in p38(MAPK) mediated apoptosis under oxidative stress. *Sci. Rep.* **7**, 15388, 10.1038/s41598-017-15342-4 (2017). 29133922 10.1038/s41598-017-15342-4 PMC5684365

[15] Gough, D. J., Koetz, L. & Levy, D. E. The MEK-ERK pathway is necessary for serine phosphorylation of mitochondrial STAT3 and Ras-mediated transformation. *PLoS ONE* **8**, e83395, 10.1371/journal.pone.0083395 (2013). 24312439 10.1371/journal.pone.0083395 PMC3843736

[16] Sang, W. et al. Expression of YAP1 and pSTAT3-S727 and their prognostic value in glioma. *J. Clin. Pathol.* **74**, 513–521, 10.1136/jclinpath-2020-206868 (2021). 33020176 10.1136/jclinpath-2020-206868

[17] Yeh, J. X., Schultz, K. L. W., Calvert, V., Petricoin, E. F. & Griffin, D. E. The NF-kappaB/leukemia inhibitory factor/STAT3 signaling pathway in antibody-mediated suppression of Sindbis virus replication in neurons. *Proc. Natl. Acad. Sci. USA* **117**, 29035–29045, 10.1073/pnas.2016691117 (2020). 33144502 10.1073/pnas.2016691117 PMC7682347

[18] Sun, S. et al. Maresin 1 mitigates sepsis-associated acute kidney injury in mice via inhibition of the NF-kappaB/STAT3/MAPK pathways. *Front. Pharm.* **10**, 1323, 10.3389/fphar.2019.01323 (2019). 10.3389/fphar.2019.01323 PMC6855000 31787899

[19] Yang, L. & Ding, J. L. MEK1/2 inhibitors unlock the constrained interferon response in macrophages through IRF1 signaling. *Front. Immunol.* **10**, 2020, 10.3389/fimmu.2019.02020 (2019). 31507609 10.3389/fimmu.2019.02020 PMC6718554

[20] Avalle, L. et al. STAT3 localizes to the ER, acting as a gatekeeper for ER-mitochondrion Ca(2+) fluxes and apoptotic responses. *Cell Death Differ.* **26**, 932–942, 10.1038/s41418-018-0171-y (2019). 30042492 10.1038/s41418-018-0171-y PMC6214529

[21] Sala, D. et al. The Stat3-Fam3a axis promotes muscle stem cell myogenic lineage progression by inducing mitochondrial respiration. *Nat. Commun.* **10**, 1796, 10.1038/s41467-019-09746-1 (2019). 30996264 10.1038/s41467-019-09746-1 PMC6470137

[22] Ziegler, P. K. et al. Mitophagy in intestinal epithelial cells triggers adaptive immunity during tumorigenesis. *Cell* **174**, 88–101.e116, 10.1016/j.cell.2018.05.028 (2018). 29909986 10.1016/j.cell.2018.05.028 PMC6354256

[23] Jhun, J. et al. GRIM19 impedes obesity by regulating inflammatory white fat browning and promoting Th17/Treg balance. *Cells* **10**, 10.3390/cells10010162 (2021). 10.3390/cells10010162 PMC7829987 33467683

[24] Kim, J. K. et al. Grim19 attenuates DSS induced colitis in an animal model. *PLoS ONE* **11**, e0155853, 10.1371/journal.pone.0155853 (2016). 27258062 10.1371/journal.pone.0155853 PMC4892643

[25] Moon, Y. M. et al. Gene associated with retinoid-interferon-induced mortality 19 attenuates murine autoimmune arthritis by regulation of th17 and treg cells. *Arthritis Rheumatol.* **66**, 569–578, 10.1002/art.38267 (2014). 24574216 10.1002/art.38267

[26] Agudo-Canalejo, J. et al. Wetting regulates autophagy of phase-separated compartments and the cytosol. *Nature* **591**, 142–146, 10.1038/s41586-020-2992-3 (2021). 33473217 10.1038/s41586-020-2992-3

[27] Kageyama, S. et al. p62/SQSTM1-droplet serves as a platform for autophagosome formation and anti-oxidative stress response. *Nat. Commun.* **12**, 16, 10.1038/s41467-020-20185-1 (2021). 33397898 10.1038/s41467-020-20185-1 PMC7782522

[28] Gassen, N. C. et al. SARS-CoV-2-mediated dysregulation of metabolism and autophagy uncovers host-targeting antivirals. *Nat. Commun.* **12**, 3818, 10.1038/s41467-021-24007-w (2021). 34155207 10.1038/s41467-021-24007-w PMC8217552

[29] Keller, C. W. et al. ATG-dependent phagocytosis in dendritic cells drives myelin-specific CD4(+) T cell pathogenicity during CNS inflammation. *Proc. Natl. Acad. Sci. USA* **114**, E11228–E11237, 10.1073/pnas.1713664114 (2017). 29233943 10.1073/pnas.1713664114 PMC5748192

[30] Romanov, J. et al. Mechanism and functions of membrane binding by the Atg5-Atg12/Atg16 complex during autophagosome formation. *EMBO J.* **31**, 4304–4317, 10.1038/emboj.2012.278 (2012). 23064152 10.1038/emboj.2012.278 PMC3501226

[31] Ji, C. & Zhao, Y. G. The BPAN and intellectual disability disease proteins WDR45 and WDR45B modulate autophagosome-lysosome fusion. *Autophagy* **17**, 1783–1784, 10.1080/15548627.2021.1924039 (2021). 34105435 10.1080/15548627.2021.1924039 PMC8354659

[32] Cai, G. et al. Discovery of fluorescent coumarin-benzo[b]thiophene 1, 1-dioxide conjugates as mitochondria-targeting antitumor STAT3 inhibitors. *Eur. J. Med. Chem.* **174**, 236–251, 10.1016/j.ejmech.2019.04.024 (2019). 31048139 10.1016/j.ejmech.2019.04.024

[33] Yang, Y. et al. Lysosomal dysfunction and autophagy blockade contribute to autophagy-related cancer suppressing peptide-induced cytotoxic death of cervical cancer cells through the AMPK/mTOR pathway. *J. Exp. Clin. Cancer Res.* **39**, 197, 10.1186/s13046-020-01701-z (2020). 32962728 10.1186/s13046-020-01701-z PMC7510096

[34] Kumsta, C. et al. The autophagy receptor p62/SQST-1 promotes proteostasis and longevity in C. elegans by inducing autophagy. *Nat. Commun.* **10**, 5648, 10.1038/s41467-019-13540-4 (2019). 31827090 10.1038/s41467-019-13540-4 PMC6906454

[35] Mauthe, M. et al. Chloroquine inhibits autophagic flux by decreasing autophagosome-lysosome fusion. *Autophagy* **14**, 1435–1455, 10.1080/15548627.2018.1474314 (2018). 29940786 10.1080/15548627.2018.1474314 PMC6103682

[36] Barrera, M. J. et al. Dysfunctional mitochondria as critical players in the inflammation of autoimmune diseases: Potential role in Sjogren’s syndrome. *Autoimmun. Rev.* **20**, 102867, 10.1016/j.autrev.2021.102867 (2021). 34118452 10.1016/j.autrev.2021.102867

[37] Zhang, H. et al. Augmenting ATG14 alleviates atherosclerosis and inhibits inflammation via promotion of autophagosome-lysosome fusion in macrophages. *Autophagy* **17**, 4218–4230, 10.1080/15548627.2021.1909833 (2021). 33849389 10.1080/15548627.2021.1909833 PMC8726734

[38] Camps, M. et al. Blockade of PI3Kgamma suppresses joint inflammation and damage in mouse models of rheumatoid arthritis. *Nat. Med.* **11**, 936–943, 10.1038/nm1284 (2005). 16127437 10.1038/nm1284

[39] Rambold, A. S. & Lippincott-Schwartz, J. Mechanisms of mitochondria and autophagy crosstalk. *Cell Cycle* **10**, 4032–4038, 10.4161/cc.10.23.18384 (2011). 22101267 10.4161/cc.10.23.18384 PMC3272286

[40] Kim, E. K. et al. IL-17-mediated mitochondrial dysfunction impairs apoptosis in rheumatoid arthritis synovial fibroblasts through activation of autophagy. *Cell Death Dis.* **8**, e2565, 10.1038/cddis.2016.490 (2017). 28102843 10.1038/cddis.2016.490 PMC5386390

[41] Mohr, A., Fahrenkamp, D., Rinis, N. & Muller-Newen, G. Dominant-negative activity of the STAT3-Y705F mutant depends on the N-terminal domain. *Cell Commun. Signal* **11**, 83, 10.1186/1478-811X-11-83 (2013). 24192293 10.1186/1478-811X-11-83 PMC3833267

[42] Saklatvala, J. Tumour necrosis factor alpha stimulates resorption and inhibits synthesis of proteoglycan in cartilage. *Nature* **322**, 547–549, 10.1038/322547a0 (1986). 3736671 10.1038/322547a0 PMC7095107

[43] Yang, L. et al. IL-21 and TGF-beta are required for differentiation of human T(H)17 cells. *Nature* **454**, 350–352, 10.1038/nature07021 (2008). 18469800 10.1038/nature07021 PMC2760130

[44] Alunno, A. et al. IL-17-producing CD4-CD8- T cells are expanded in the peripheral blood, infiltrate salivary glands and are resistant to corticosteroids in patients with primary Sjogren’s syndrome. *Ann. Rheum. Dis.* **72**, 286–292, 10.1136/annrheumdis-2012-201511 (2013). 22904262 10.1136/annrheumdis-2012-201511

[45] Chen, Y. M. et al. Association between autophagy and inflammation in patients with rheumatoid arthritis receiving biologic therapy. *Arthritis Res. Ther.* **20**, 268, 10.1186/s13075-018-1763-0 (2018). 30518408 10.1186/s13075-018-1763-0 PMC6280483

[46] Schrezenmeier, E. & Dorner, T. Mechanisms of action of hydroxychloroquine and chloroquine: implications for rheumatology. *Nat. Rev. Rheumatol.* **16**, 155–166, 10.1038/s41584-020-0372-x (2020). 32034323 10.1038/s41584-020-0372-x

[47] Sonn, S. K. et al. ER-associated CTRP1 regulates mitochondrial fission via interaction with DRP1. *Exp. Mol. Med.* **53**, 1769–1780, 10.1038/s12276-021-00701-z (2021). 34837016 10.1038/s12276-021-00701-z PMC8639813

[48] Galvan, D. L. et al. Drp1S600 phosphorylation regulates mitochondrial fission and progression of nephropathy in diabetic mice. *J. Clin. Invest*. **129**, 2807–2823, 10.1172/JCI127277 (2019). 31063459 10.1172/JCI127277 PMC6597204

[49] Cloonan, S. M. & Choi, A. M. Mitochondria in lung disease. *J. Clin. Invest.* **126**, 809–820, 10.1172/JCI81113 (2016). 26928034 10.1172/JCI81113 PMC4767339

[50] Clayton, S. A., MacDonald, L., Kurowska-Stolarska, M. & Clark, A. R. Mitochondria as key players in the pathogenesis and treatment of rheumatoid arthritis. *Front. Immunol.* **12**, 673916, 10.3389/fimmu.2021.673916 (2021). 33995417 10.3389/fimmu.2021.673916 PMC8118696

[51] Rincon, M. & Pereira, F. V. A new perspective: mitochondrial stat3 as a regulator for lymphocyte function. *Int. J. Mol. Sci*. **19**, 10.3390/ijms19061656 (2018). 10.3390/ijms19061656 PMC6032237 29866996

[52] Kim, D. Y., Lim, S. G., Suk, K. & Lee, W. H. Mitochondrial dysfunction regulates the JAK-STAT pathway via LKB1-mediated AMPK activation ER-stress-independent manner. *Biochem. Cell Biol.* **98**, 137–144, 10.1139/bcb-2019-0088 (2020). 31071273 10.1139/bcb-2019-0088

[53] Poholek, C. H. et al. Noncanonical STAT3 activity sustains pathogenic Th17 proliferation and cytokine response to antigen. *J. Exp. Med.* **217**, 10.1084/jem.20191761 (2020). 10.1084/jem.20191761 PMC7537401 32697822

[54] Yang, R. & Rincon, M. Mitochondrial Stat3, the need for design thinking. *Int. J. Biol. Sci.* **12**, 532–544, 10.7150/ijbs.15153 (2016). 27019635 10.7150/ijbs.15153 PMC4807418

[55] Yu, H., Pardoll, D. & Jove, R. STATs in cancer inflammation and immunity: a leading role for STAT3. *Nat. Rev. Cancer* **9**, 798–809, 10.1038/nrc2734 (2009). 19851315 10.1038/nrc2734 PMC4856025

[56] Zimmers, T. A., Fishel, M. L. & Bonetto, A. STAT3 in the systemic inflammation of cancer cachexia. *Semin. Cell Dev. Biol.* **54**, 28–41, 10.1016/j.semcdb.2016.02.009 (2016). 26860754 10.1016/j.semcdb.2016.02.009 PMC4867234

[57] Sherlock, J. P. et al. IL-23 induces spondyloarthropathy by acting on ROR-gammat+ CD3+CD4-CD8- entheseal resident T cells. *Nat. Med.* **18**, 1069–1076, 10.1038/nm.2817 (2012). 22772566 10.1038/nm.2817

[58] Chen, B. et al. Cellular zinc metabolism and zinc signaling: from biological functions to diseases and therapeutic targets. *Signal Transduct. Target Ther.* **9**, 6, 10.1038/s41392-023-01679-y (2024). 38169461 10.1038/s41392-023-01679-y PMC10761908

[59] Comita, S. et al. Regulation of STAT3 and its role in cardioprotection by conditioning: focus on non-genomic roles targeting mitochondrial function. *Basic Res. Cardiol.* **116**, 56, 10.1007/s00395-021-00898-0 (2021). 34642818 10.1007/s00395-021-00898-0 PMC8510947

[60] Zhang, G. et al. Zinc improves mitochondrial respiratory function and prevents mitochondrial ROS generation at reperfusion by phosphorylating STAT3 at Ser(727). *J. Mol. Cell Cardiol.* **118**, 169–182, 10.1016/j.yjmcc.2018.03.019 (2018). 29605530 10.1016/j.yjmcc.2018.03.019

