# Supplementary material for: mtSTAT3 suppresses rheumatoid arthritis by regulating Th17 and synovial fibroblast inflammatory cell death with IL-17-mediated autophagy dysfunction
Source: Exp Mol Med. 2025 Jan 17;57(1):221–34. doi: 10.1038/s12276-024-01376-y (PMC11799179; doi:10.1038/s12276-024-01376-y)
Supplement: Supplementary file 1 — Supplementary Information [file 12276_2024_1376_MOESM1_ESM.pdf]

Supplementary Methods

Construction of plasmid vector

The MLS-STAT3 overexpression vector was generated by GenScript Corporation (Piscataway, NJ, USA). The MLS-STAT3 vector construct tagged with FLAG was inserted into the BamHI and NotI sites of the pEGFP-N3 vector and designated pEG-MLS-STAT3-FLAG. The pRc/CMV vector carrying mouse Stat3^{Y705F} (STAT3 Y705F, in which tyrosine 705 is replaced with a phenylalanine residue) and FLAG was generously provided by Dr. Jim Darnell (Addgene plasmid #8709). The plasmids were produced by Macrogen, Inc.

Electron microscopy imaging

RA FLS and OA FLS cells were fixed in 4% paraformaldehyde and 2.5% glutaraldehyde in 0.1 M phosphate buffer overnight at 4°C. The cells were washed in 0.1 M phosphate buffer, postfixed with 1% osmium tetroxide for 1h at 4°C, dehydrated in graded ethyl alcohol solutions, exchanged in acetone, and embedded in Epon 812. Ultrathin sections (70–80 nm) were obtained on an ultramicrotome (Leica) and stained with uranyl acetate and lead citrate. Images were acquired at 60 kV using a transmission electron microscope (TEM).

Induction of collagen antibody-induced arthritis

To induce arthritis, C57BL/6 mice were intravenously administered a cocktail of monoclonal antibodies to type II collagen (Chondrex). On day 3 after the monoclonal antibody injection,

we intraperitoneally injected LPS 0114:B4 (Sigma-Aldrich Co.) to stimulate induction of RA.

MitoSTAT3 transfection

In total, 2 × 10^5 RA fibroblast-like synoviocytes (FLSs) were transfected with Mock or MLS-STAT3 DNA vector. Transfection of Mock and MLS-STAT3 DNA vectors was performed using the X-tremeGENE™ HP DNA Transfection Reagent (Roche) in accordance with the manufacturer's recommendations. The transfection medium of the RA FLSs was replaced at 6 h post-transfection, and the cells were cultured with recombinant human IL-17 (R&D Systems Inc.) for 24h. The cells were analyzed for autophagosome and autophagolysosome. The isolated cell lysates were used for western blot analysis.

Culture of RA FLSs and mitochondrial isolation

RA FLSs (5 × 10^5 cells) were cultured with IL-17 (2, 5, 10, or 50 ng/mL) for 12 h. Cytosolic and mitochondrial fractions were extracted from cells using a mitochondrial isolation kit (Thermo Fisher Scientific) for western blot analysis.

Th17 cell differentiation

Splenocytes were cultured with stimulators of Th17 cell differentiation. The splenocytes were stimulated with 0.5 μg/mL anti-mouse CD3 (BD Biosciences), 1 μg/mL anti-mouse CD28 (BD Biosciences), 5 μg/mL anti-mouse IFN-γ (BioLegend), 5 μg/mL anti-mouse IL-4 (BioLegend), 1 ng/mL transforming growth factor-β (PeproTech), and 20 ng/mL IL-6 (R&D Systems) with or without 10 μM $ZnSO_4$ (Sigma-Aldrich) for 3 days.

Supplementary Figures

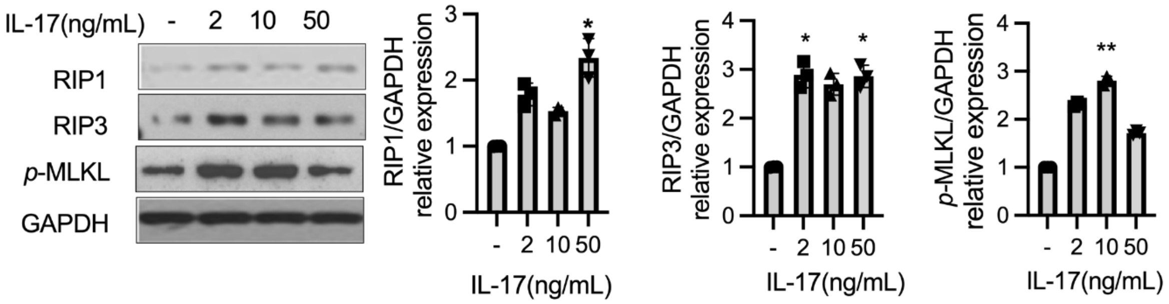

Supplementary Fig. 1. IL-17 increased inflammatory cell death in RA FLSs

RA FLSs (5×10^5 cells) were cultured with IL-17 (2, 10, or 50 ng/mL) for 12 h followed by lysis to acquire proteins. RIP1, RIP3, and *p*-MLKL were detected by western blot analysis using antibodies from the respective cell lysates. Expression levels of RIP1, RIP3, and *p*-MLKL are displayed.

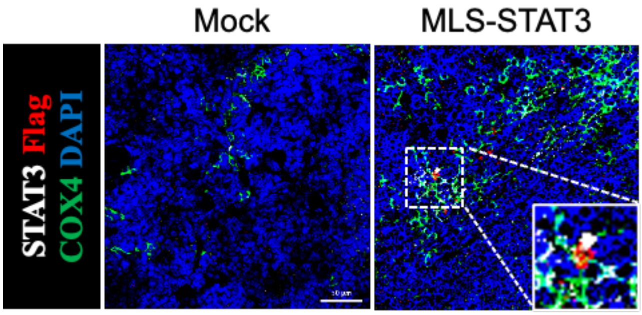

Supplementary Fig. 2. The increased expression of mitochondrial STAT3 was confirmed by in the spleen tissue

The immunofluorescence images of splenic tissues were stained with anti-STAT3, anti-Flag, anti-COX4 antibodies and DAPI. Scale Bar = 50μm.

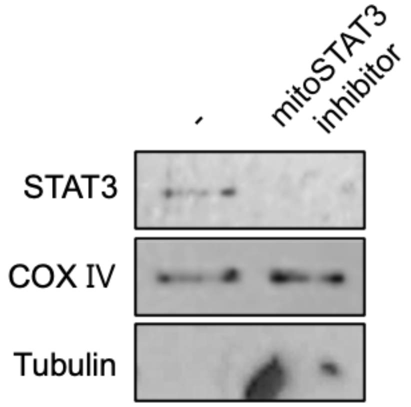

Supplementary Fig. 3. mitoSTAT3 inhibitor inhibits the STAT3 expression in mitochondria of OA FLS

The 1 μM of mitoSTAT3 inhibitor was treated on the OA FLS including 20 ng/ml of IL-17. Then, mitochondria were isolated and western blotting was performed with anti-STAT3, anti-COX4 and anti-tubulin antibodies.

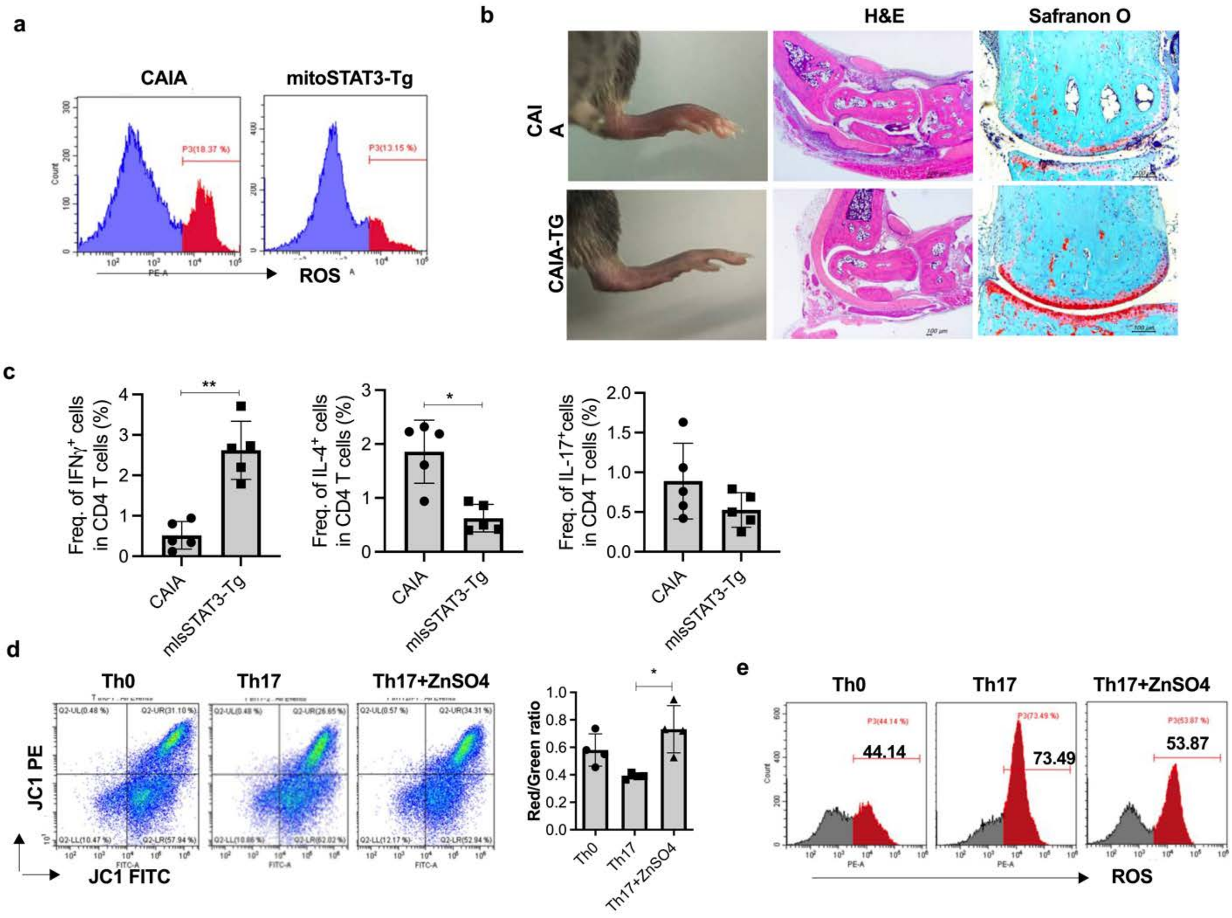

Supplementary Fig. 4. mitoSTAT3 inhibited mitochondrial ROS, Th17 development, and arthritis induction

a, Mitochondrial ROS were measured by flow cytometry of splenocytes from mitoSTAT3 Tg mice. **b,** 4 weeks after the first immunization, ankle joint tissues were obtained from each group, and the sectioned tissues were stained with H&E and safranin O. **c,** Th1, Th2, and Th17 cells were analyzed by flow cytometry of splenocytes from mitoSTAT3 Tg mice. **d and e**, Splenic CD4$^+$ T cells from WT mice were cultured under Th17 cell-inducing conditions in the presence or absence of zinc sulfate (10 μM). Mitochondrial membrane potentials and mitochondrial ROS were measured using JC-1 or MitoSOX dye and analyzed using flow cytometry. The bar graph is presented as mean ± standard deviation (*p < 0.05, **p < 0.01).

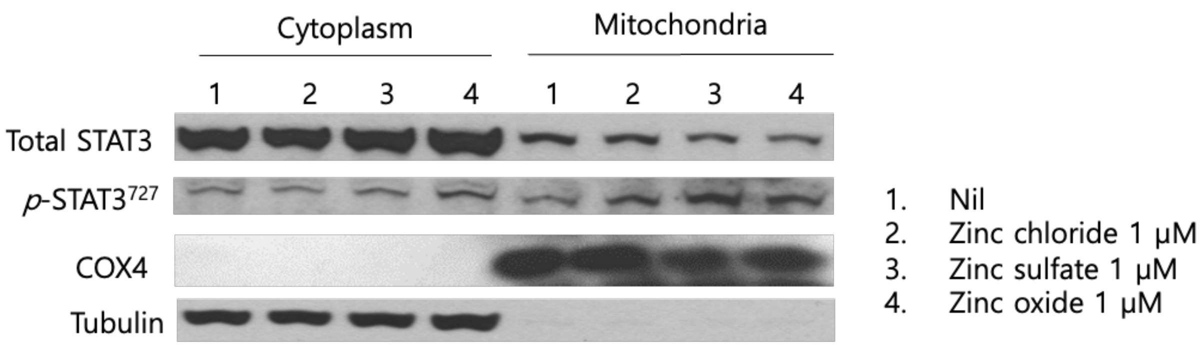

Supplementary Fig. 5. Zinc compounds increased mitochondrial STAT3 in murine splenocytes

The splenocytes (5×10^5 cells) were cultured with 1 μM of zinc chloride, zinc sulfate and zinc oxide respectively. Cell lysate was collected separately into cytosol and mitochondria. Then, western blot was performed using antibodies against total STAT3, *p*-STAT3^{Ser727}, COX4 and tubulin, respectively.
